# Deep Learning-based Alignment Measurement in Knee Radiographs

**DOI:** 10.1007/978-3-032-04965-0_12

**Published:** 2025-09-19

**Authors:** Zhisen Hu, Dominic Cullen, Peter Thompson, David Johnson, Chang Bian, Aleksei Tiulpin, Timothy Cootes, Claudia Lindner

**Affiliations:** 1 Division of Informatics, Imaging and Data Sciences, The University of Manchester, United Kingdom; 2 Research Unit of Health Sciences and Technology, University of Oulu, Finland; 3 Northern Care Alliance NHS Foundation Trust, United Kingdom; 4 Department of Trauma and Orthopaedics, Stockport NHS Foundation Trust, Stepping Hill Hospital, United Kingdom; 5 School of Health and Society, University of Salford, United Kingdom; 6 School of Biological Sciences, The University of Manchester, United Kingdom

**Keywords:** Knee alignment, Landmark localization, Deep learning, Hourglass, Anatomical tibiofemoral angle

## Abstract

Radiographic knee alignment (KA) measurement is important for predicting joint health and surgical outcomes after total knee replacement. Traditional methods for KA measurements are manual, time-consuming and require long-leg radiographs. This study proposes a deep learning-based method to measure KA in anteroposterior knee radiographs via automatically localized knee anatomical landmarks. Our method builds on hourglass networks and incorporates an attention gate structure to enhance robustness and focus on key anatomical features. To our knowledge, this is the first deep learning-based method to localize over 100 knee anatomical landmarks to fully outline the knee shape while integrating KA measurements on both pre-operative and post-operative images. It provides highly accurate and reliable anatomical varus/valgus KA measurements using the anatomical tibiofemoral angle, achieving mean absolute differences ~1° when compared to clinical ground truth measurements. Agreement between automated and clinical measurements was excellent pre-operatively (intra-class correlation coefficient (ICC) = 0.97) and good post-operatively (ICC = 0.86). Our findings demonstrate that KA assessment can be automated with high accuracy, creating opportunities for digitally enhanced clinical workflows.

## 1 Introduction

Knee osteoarthritis (OA) is a common and significant health issue that heavily burdens healthcare systems [1]. Total knee replacement (TKR) may be offered as treatment for end-stage knee OA. Nevertheless, TKR is invasive involving prosthesis implantation at the knee joint, and around 10% of patients are dissatisfied following TKR [2, 3]. Pre-operative and post-operative knee alignment (KA) affects the outcomes following TKR, with radiographs revealing anomalies such as deformities of the femur and tibia, as well as incorrect positioning of the implants [4, 5]. Accurate assessment of KA in radiographs is important for successful treatment outcomes and long-term joint health. Traditional KA measurement methods are manual, time-consuming, and require long-leg radiographs. However, long-leg radiographs are not always undertaken in clinical practice, and standard anteroposterior (AP) knee radiographs are often the main imaging modality. Automated methods for measuring KA in AP knee radiographs are potentially clinically valuable for reducing the cost and improving the efficiency of the knee OA treatment pathway.

**Fig. 1:**
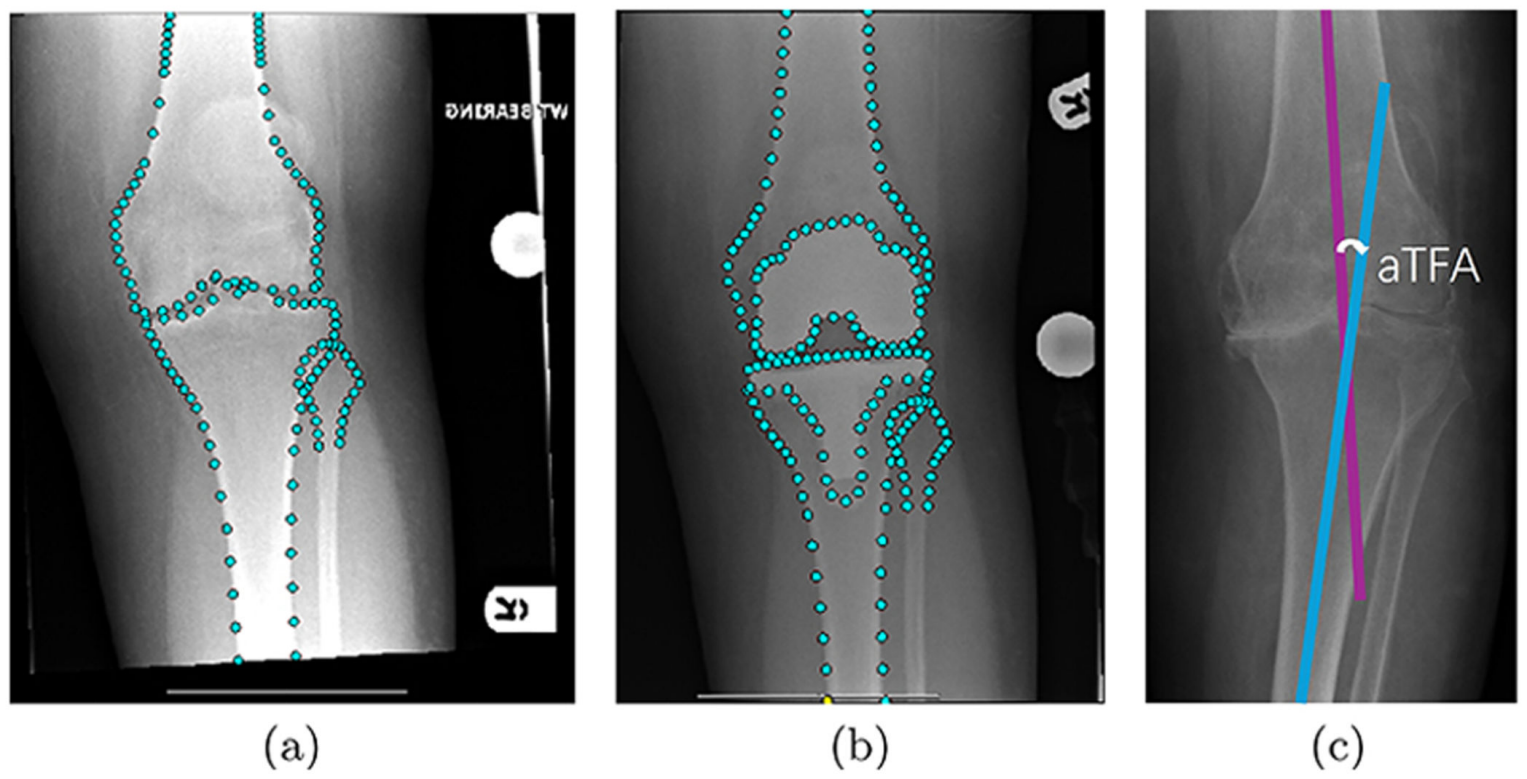
An illustration of AP knee radiographs with corresponding anatomical landmarks in (a) pre-operative and (b) post-operative images, and (c) the anatomical tibiofemoral angle (aTFA) measurement. aTFA is defined by the angle between the anatomical femoral axis (purple line) and tibial axis (blue line).

Knee anatomical landmark positions (Fig. 1a and 1b) are often used for automatically generating KA measurements [17, 18]. Recently, machine learning and deep learning have been widely used for localizing knee anatomical landmarks in radiographs. One of the state-of-the-art methods of knee landmark localization is based on a combination of random forest regression voting (RFRV) with constrained local model (CLM) fitting [6, 7]. In [13], this RFRV-CLM framework was effectively applied to localize key knee landmark positions for anatomical tibiofemoral angle (aTFA) measurement, marking a significant advancement in automated KA assessment. State-of-the-art deep learning-based methods include the study by Tiulpin et al. [8], which used hourglass networks [10] to regress the knee landmark positions from AP knee radiographs. Several other methods used U-Nets [14] to localize pelvis and hand landmarks [15, 16]. The landmark localization stage in our method is based on the hourglass network architecture in [8] and combines the network with an attention gate (AG) structure [9] to better focus on target joint shapes in knee radiographs.

This study proposes a deep learning-based approach to automatically localize knee anatomical landmarks and measure varus and valgus KA using the aTFA. In both pre-operative and post-operative AP knee radiographs, the aTFA is defined by the angle between the anatomical femoral and tibial axes (Fig. 1c). To our knowledge, this is the first deep learning-based study to localize over 100 anatomical landmarks in knee radiographs and integrate KA measurements on both pre-operative and post-operative images.

### Contributions

1) We compare our method with the approach presented in [13], demonstrating superior accuracy in knee landmark localization and improved overall performance in KA measurements across both pre-operative and post-operative knee radiographs.
2) We further investigate how different strategies for generating KA measurements, specifically the use of different subsets of landmark positions, influence the level of agreement with ground truth measurements. This evaluation highlights the impact of landmark selection on measurement reliability and clinical relevance.

## 2 Method

**Fig. 2:**
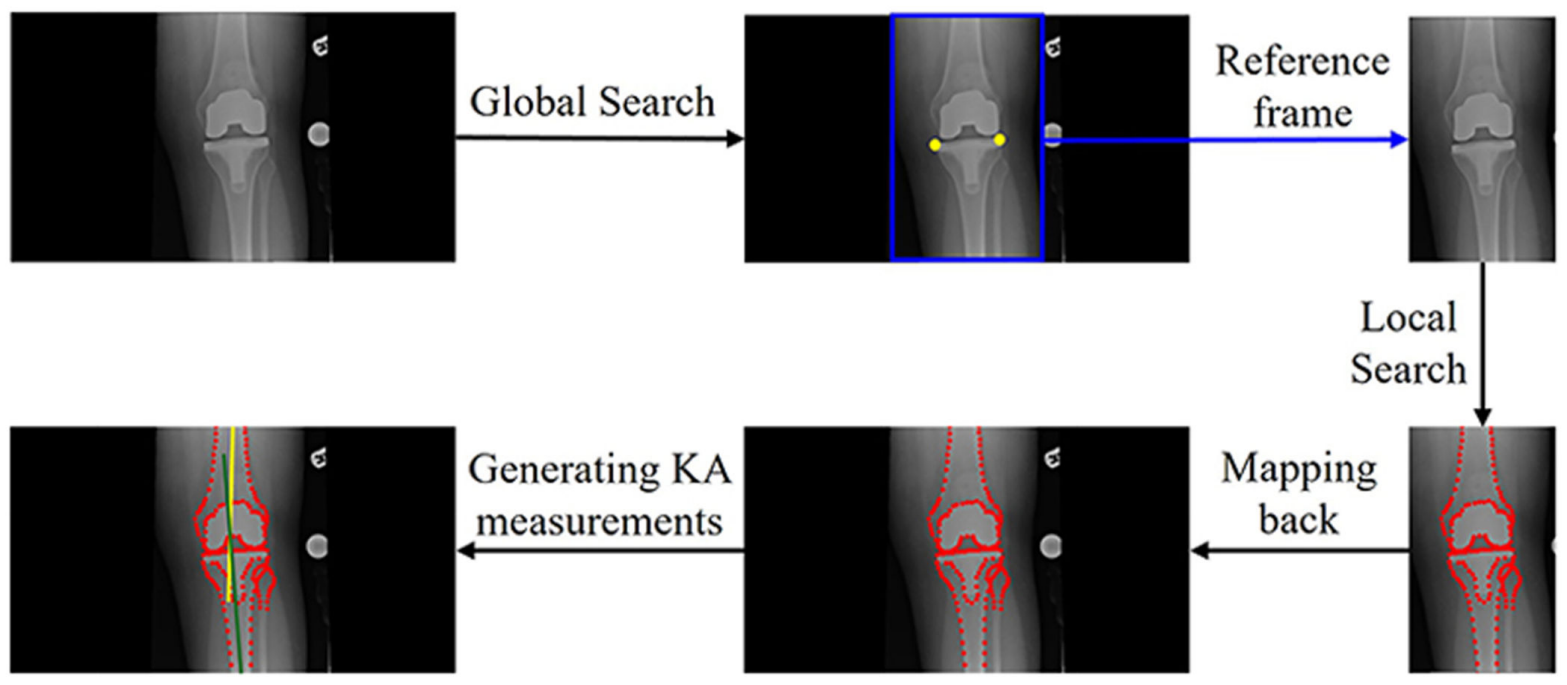
The workflow of our automated KA measurement approach. The global search model searches across the entire image and locates two reference points (yellow points), which establish the approximate position, orientation, and scale of a reference frame (region of interest). Then the local search model finds over 100 knee landmarks (red points) within the reference frame to outline the shape of the knee joint. The landmarks are mapped back to the original image for comparison with the original manual annotations. The KA measurements are then generated from these landmark positions.

The workflow of our automated KA measurement approach is shown in Fig. 2. The knee landmarks are localized first, and subsequently the KA measurements are generated based on the landmark positions.

### 2.1 Data

Our dataset consists of anonymized standard AP knee radiographs from TKR patients. To simplify the analysis, all right knee radiographs were flipped horizontally to appear as left knee radiographs. All radiographs were retrospectively collected from Stockport NHS Foundation Trust (approved by the Health Research Authority, IRAS 244130). All subjects underwent primary TKR and had no revision surgery within three years after TKR. Our dataset consists of 566 pre-operative and 457 one-year post-operative images for training, and 376 patient image pairs (pre-operative and one-year post-operative) for testing [13, 21]. In the test set, 58 participants (15.4%) had unknown gender or ethnicity, and 2 (0.5%) had unknown age. Among cases with complete demographic information, the mean age was 69.2 ± 8.7 years, with 42.1% identified as male and 88.4% as White. As TKR primarily affects older adults, this age distribution aligns with real-world patient demographics. Landmarks were defined along the distal femur and proximal tibia/fibula to capture the knee joint, including implants in the post-operative images (see Fig. 1a and 1b). The pre-operative and post-operative images were manually annotated with 134 and 181 landmarks, respectively.

### 2.2 Landmark Localization

**Fig. 3:**
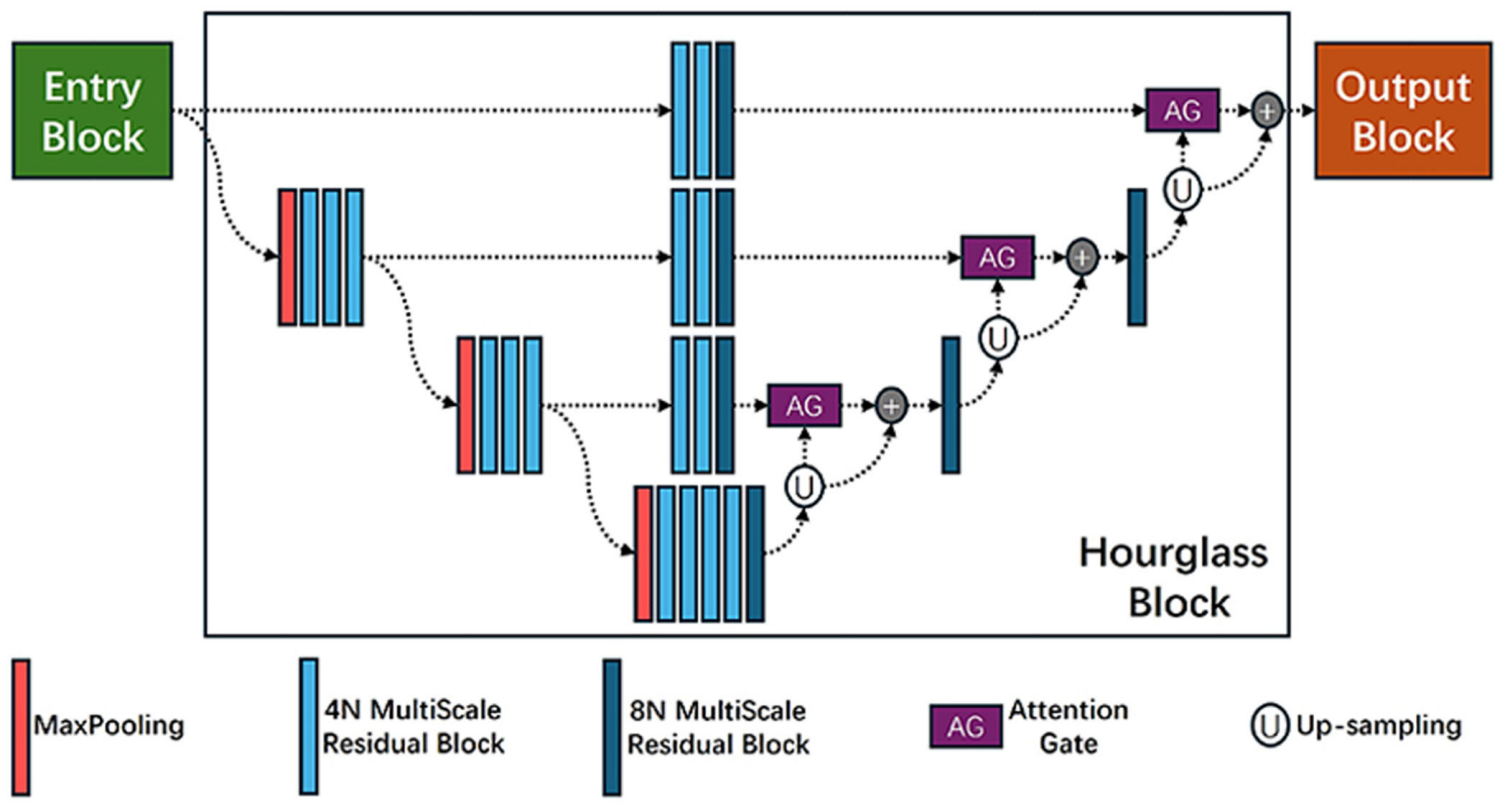
Model architecture with an hourglass network of depth d=4 combined with AGs. Here, N is the width (initial number of channels) of the network.

A deep learning-based system using hourglass networks was trained to localize the anatomical knee landmarks. Our network structure (shown in Fig. 3) is similar to the hourglass network in [8]. AG blocks similar to [9] are used to filter the features passed from the upper-level blocks of the hourglass network. Our automated landmark localization system consists of two stages: global search and local search (with an independent hourglass network for each stage).

The global search aims to narrow down the search area for the subsequent local search stage. We use a 4-layer hourglass network to scan the entire knee radiograph to identify two reference points on the knee joint. In our case, we chose two landmarks of the local search model for the purpose of initialization. The two reference points have a fixed position in the reference frame, which is centered around the region of interest identified in the global search. The position, orientation, and scale of the reference frame are defined by the two reference points.

The local model searches in the more confined reference frame. The objective is to accurately localize specific landmarks on the target object. We use a 6-layer hourglass network to localize the knee landmarks. The landmark positions are then mapped back to the original image using the position, orientation, and scale obtained from the global search.

### 2.3 Knee Alignment

We measured varus/valgus KA in standard AP knee radiographs both pre-operatively and post-operatively using the aTFA (Fig. 1c). Varus and valgus were defined as negative and positive deviations from zero, respectively. We included two sets of point-based measurements in our experiments. **Automated measurements** were assessed based on a subset of the *automatically localized* pre-operative and post-operative landmark positions in the 376 test patients. **Manual measurements** were generated based on a subset of the *manually annotated* landmark positions in pre-operative and post-operative images of the 376 test patients and were used as the manual ground-truth.

In addition, we also included a set of **clinical measurements** which were directly measured in a clinical setting with a Picture Archiving and Communication System (PACS)-integrated measurement facility by an orthopedic surgeon. Clinical measurements were obtained for a random subset of 50 test patients from the 376 test patients. Two clinical measurements were taken for each image, with a 7-10 day interval between them, and the second measurement was made without knowledge of the first. The mean of the two measurements was used as the clinical ground truth.

**Fig. 4:**
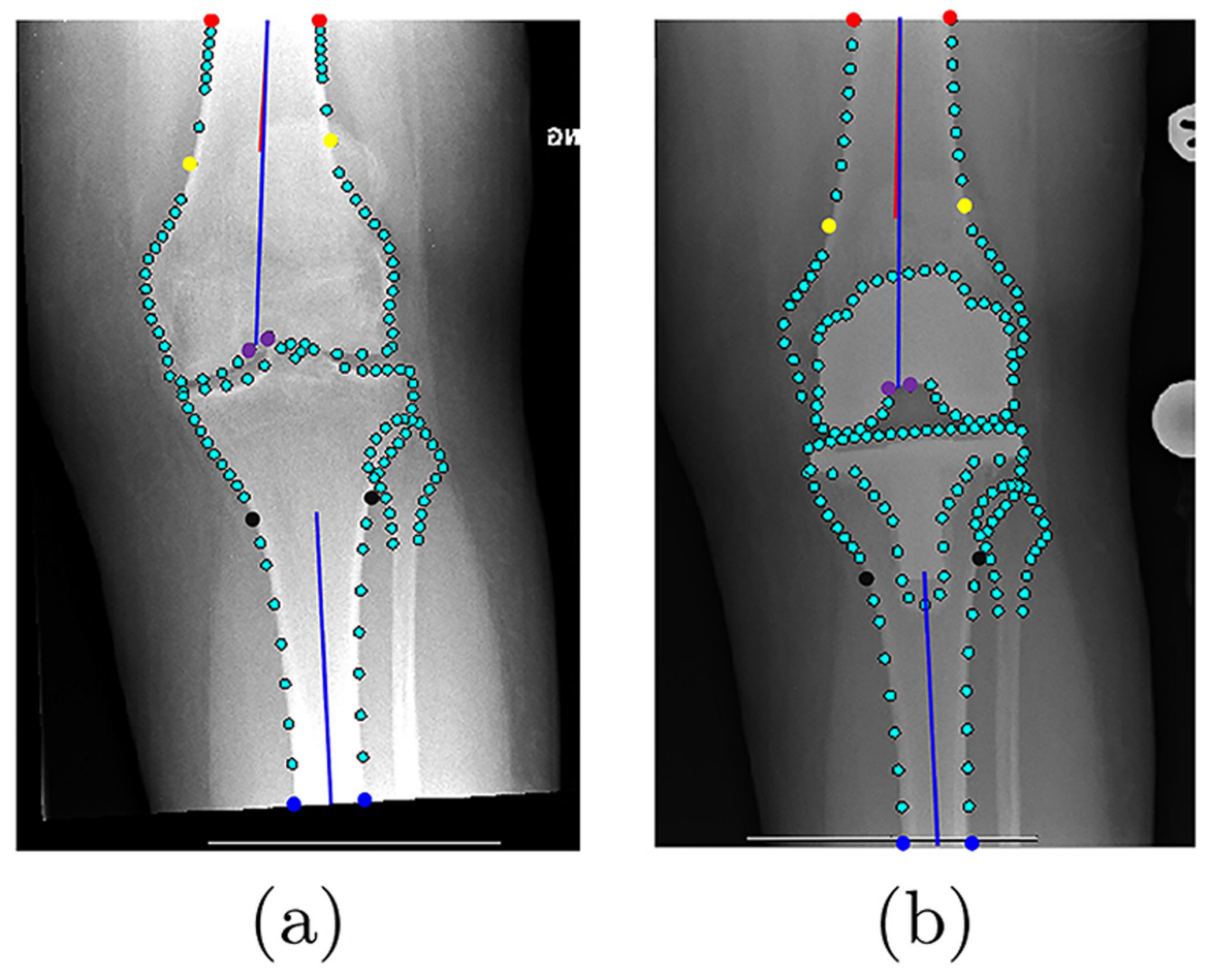
An illustration of the two calculation methods of the point-based KA measurements in (a) pre-operative and (b) post-operative images. In both (a) and (b), FTS fits a red center line to the femur and tibia by connecting two shaft center points (femur: the mid-points of the red and yellow point pairs; tibia: the mid-points of the black and blue point pairs), whereas FNTS fits a blue line to the femur by connecting a shaft center point (mid-point of the red point pair) to a femoral notch point (mid-point of the purple point pair), and to the tibia by connecting two shaft center points (mid-points of the black and blue point pairs). The blue lines may overlap the red lines.

We investigated two calculation methods for the point-based measurements: one (**FTS**) using only femoral and tibial shaft points, and another (**FNTS**) incorporating femoral notch information with the femoral and tibial shaft points. The two calculation methods in pre-operative and post-operative knee radiographs are visualized in Fig. 4a and 4b, respectively.

## 3 Experiments

### 3.1 Implementation Details

Hourglass networks were trained using PyTorch 2.3.1 for deep learning-based pre-operative and post-operative knee radiograph analysis, with 220 and 120 epochs for global search and 800 and 600 epochs for local search, respectively. Global search models were trained on NVIDIA Tesla V100 GPUs, while local search models were trained on NVIDIA A100 GPUs. The network widths (initial numbers of channels) were 32 and 256 for global and local search, respectively. Wing loss [11] was applied to emphasize small errors, and the model was optimized with Adam [12] using a learning rate of 0.0001.

### 3.2 Results

#### Landmark Localization

We evaluated our landmark localization approach using relative point-to-point (rP2P) and relative point-to-curve (rP2C) distances. P2P is the Euclidean distance between a predicted and manual ground-truth point, and P2C is the distance between a predicted point to the bone boundary based on the ground truth points. Both metrics are computed per point and averaged across all points within an image. The relative distances were calculated to show the percentage of the reference length (tibial shaft width) defined by the distance between the two landmarks at the corners of the tibial plateau (see yellow points in Fig. 2). We had access to the pre-operative and post-operative RFRV-CLM models from [13] to compare the landmark detection accuracy with our approach. The results are summarized in Table 1. We found that our hourglass-based method could localize the knee landmarks more accurately and robustly than [13] with lower rP2P and rP2C distances.

#### Alignment Measurement

Intra-class corelation coefficient (ICC), mean absolute difference (MAD), and Bland-Altman analysis (BAA) were used to assess the agreement between the automated measurements generated from the automatically localized landmark positions and the two sets of ground truth measurements. Higher ICC and lower MAD or BAA bias indicate better performance. In Table 2, we summarize the results of our KA measurement experiments, and compare the results to those presented in [13].

*FTS* When analyzing the agreement between manual/clinical and automated measurements (Table 2), the pre-operative ICC values showed excellent agreement (>0.9), whereas the post-operative ICC values showed good agreement (0.75-0.9). The MAD values indicated minimal deviations (~1°) pre-operatively and post-operatively. The BAA showed no bias (<1°). Our method outper-formed [13] in most cases, except for the post-operative ICC value between manual and automated measurements.

*FNTS* When analyzing the agreement between manual/clinical and automated measurements (Table 2), only the post-operative ICC value between clinical and automated measurements showed good agreement (0.75-0.9), while other ICC values showed excellent agreement (>0.9). The MAD values indicated minimal deviations (~1°) pre-operatively and post-operatively. The BAA showed no bias (<1°). Our method outperformed [13] with a higher ICC value, as well as lower MAD value and similar BAA bias.

In most cases, FNTS demonstrated better agreement than FTS except for the post-operative agreement between clinical and automated measurements.

## 4 Discussion and Conclusion

We developed an automated system to localize knee anatomical landmarks and measure KA. To our knowledge, this is the first deep learning-based system to localize over 100 knee landmarks, fully outlining the knee joint while integrating KA measurements on AP knee radiographs. Our results on pre-operative and post-operative images from 376 TKR patients show that our hourglass-based system achieves consistently improved performance in localization accuracy compared with [13].

The system demonstrates excellent accuracy and reliability in measuring varus/valgus KA. Our method achieves better performance than [13] except for the post-operative ICC value between manual and automated measurements when calculating the aTFA with FTS. Post-operative agreement is lower than pre-operative agreement in terms of ICC, especially when comparing clinical and automated measurements, likely due to anatomical changes from TKR not captured well by our point-based definitions. The automated measurements show a higher agreement with the manual measurements compared to the clinical measurements, possibly because of additional considerations in clinical practice like limb deformities instead of only using point position-based information. When calculating the aTFA, incorporating the femoral notch information can improve the overall reliability except when assessing the post-operative agreement between clinical and manual measurements.

As clinical measurements of only a single expert were used as reference in this study, additional clinical measurements should be added to analyze the clinical variation in the future. A limitation of this study is that the system has not been tested for generalizability on another dataset. It would be of interest to use our trained models to generate KA measurements on an independent dataset.

KA is strongly associated with TKR outcomes. For example, both varus and valgus post-operative malalignment were found to be associated with a higher incidence of revision surgery in several studies [19, 4, 5]. Future work will explore the relationship between KA measurements and TKR outcomes, aiming to predict surgical outcomes such as chronic pain or revision surgery in advance based on KA measurements in knee radiographs. In addition, the automatically localized landmark positions enable more complex analysis of knee joint shape and alignment (e.g. via Statistical Shape Models [20]), beyond what can be currently captured by a set of geometric measurements. This opens up opportunities for better use of the information contained in AP knee radiographs, enabling more efficient and appropriate treatment decisions.

## Figures and Tables

**Table 1:** Quantitative comparison of pre-operative and post-operative landmark localization accuracy in AP knee radiographs between the proposed method and [13]. The relative point-to-point (rP2P) and relative point-to-curve (rP2C) distances were calculated to show the percentage of the reference length (tibial shaft width) defined by the distance between the two landmarks at the corners of the tibial plateau (see yellow points in Fig. 2).

Data	Method	rP2P			rP2C		
		Mean	Median	95%ile	Mean	Median	95%ile
Pre-operative	RFRV-CLM [13]	4.1%	3.4%	9.5%	1.1%	0.6%	2.1%
	**This study**	**1.7**%	**1.6**%	**2.5**%	**0.6**%	**0.5**%	**0.8**%
Post-operative	RFRV-CLM [13]	3.6%	2.5%	11.7%	1.3%	0.6%	9.0%
	**This study**	**1.6**%	**1.6**%	**2.4**%	**0.4**%	**0.4**%	**0.6**%

**Table 2:** Agreement between automated and manual/clinical aTFA measurements calculated by **FTS** and **FNTS**. The best performances, compared with clinical measurements, are highlighted with *. (Pre-op: Pre-operative; Post-op: Post-operative; M: manual; A: automated; C: clinical; ICC: intra-class correlation coefficient; MAD: mean absolute difference; BAA: Bland-Altman analysis; n: number of individuals; CI: confidence interval; SD: standard deviation)

aTFA	Method	Agreement	ICC		MAD		BAA	
			Value	CI 95%	Value	SD	Bias	SD
FTS	RFRV- CLM [13]	Pre-op M and A (n=376)	0.97	(0.96, 0.97)	1.0°	1.3°	0.2°	1.6°
		Post-op M and A (n=376)	**0.88**	(0.86, 0.90)	0.9°	1.1°	0.2°	1.4°
		Pre-op C and A (n=50)	**0.95**	(0.91, 0.97)	1.4°	1.4°	**-0.8°**	1.8°
		Post-op C and A (n=50)	0.78	(0.67, 0.86)	1.5°	1.3°	0.5°	2.0°
	This study	Pre-op M and A (n=376)	**0.99**	(0.99, 0.99)	**0.6°**	0.7°	**0.1°**	1.0°
		Post-op M and A (n=376)	0.83	(0.80, 0.86)	**0.6°**	1.6°	**0.0°**	1.7°
		Pre-op C and A (n=50)	**0.95**	(0.91, 0.98)	**1.3°**	1.3°	**-0.8°**	1.7°
		Post-op C and A (n=50)*	**0.86**	(0.76, 0.92)	**1.0°**	1.3°	**0.2°**	1.6°
FNTS	RFRV- CLM [13]	Pre-op M and A (n=376)	0.98	(0.97, 0.98)	0.8°	1.1°	0.2°	1.4°
		Post-op M and A (n=376)	0.92	(0.90, 0.93)	0.8°	0.8°	**0.1°**	1.1°
		Pre-op C and A (n=50)	**0.97**	(0.95, 0.98)	**1.2°**	1.0°	**0.1°**	1.6°
		Post-op C and A (n=50)	0.71	(0.56, 0.81)	1.7°	1.5°	0.8°	2.2°
	This study	Pre-op M and A (n=376)	**0.99**	(0.99, 0.99)	**0.6°**	0.6°	**0.0°**	0.8°
		Post-op M and A (n=376)	**0.96**	(0.95, 0.97)	**0.5°**	0.6°	**0.1°**	0.7°
		Pre-op C and A (n=50)*	**0.97**	(0.95, 0.98)	**1.2°**	1.0°	**0.1°**	1.6°
		Post-op C and A (n=50)	**0.81**	(0.69, 0.89)	**1.2°**	1.4°	**0.5°**	1.7°
